# Paeoniflorin inhibits the macrophage-related rosacea-like inflammatory reaction through the suppressor of cytokine signaling 3-apoptosis signal-regulating kinase 1-p38 pathway

**DOI:** 10.1097/MD.0000000000023986

**Published:** 2021-01-22

**Authors:** Zijing Liu, Jiawen Zhang, Peiyu Jiang, Zhi Yin, Yunyi Liu, Yixuan Liu, Xiaoyan Wang, Liang Hu, Yang Xu, Wentao Liu

**Affiliations:** a Department of Dermatology, the First Affiliated Hospital of Nanjing Medical University, Nanjing, China; b Department of Pharmacology, Nanjing Medical University, Nanjing, Jiangsu, People's Republic of China.

**Keywords:** apoptosis signal-regulating kinase 1, macrophage, paeoniflorin, rosacea, suppressor of cytokine signaling 3

## Abstract

Rosacea is a facial chronic inflammatory skin disease with immune and vascular system dysfunction. Paeoniflorin (PF) is a traditional Chinese medicine with anti-inflammatory properties. However, its effects on rosacea remain unknown. Here, we investigated the mechanisms through which PF inhibits the macrophage-related rosacea-like inflammatory response. Immunohistochemical methods were used to detect differences in the inflammatory response and degree of macrophage infiltration in granulomatous rosacea lesions and their peripheral areas. Cell Counting Kit-8 was used to determine the cytotoxicity of PF towards RAW 264.7 cells. Reverse transcription-quantitative polymerase chain reaction and western blotting were used to measure the influence of PF on mRNA and protein expression levels of suppressor of cytokine signaling 3 (SOCS3), apoptosis signal-regulating kinase 1 (ASK1)-p38, Toll-like receptor 2, and cathelicidin antimicrobial peptide ( or LL37) in the lipopolysaccharide (LPS)-induced macrophage-related rosacea-like inflammatory response of RAW 264.7 cells. Inflammatory cell infiltration was more pronounced in granulomatous rosacea lesions than in peripheral areas. LL37 expression increased significantly, and the infiltration of a large number of CD68^+^ macrophages was observed in the lesions. PF promoted SOCS3 expression in RAW 264.7 cells and inhibited the LPS-induced increase in toll-like receptor 2 and LL37 expression through the ASK1-p38 cascade, thereby alleviating the macrophage-related rosacea-like inflammatory response. These changes could be abrogated by SOCS3 siRNA in vitro.

In conclusion, the pathogenesis of rosacea involves abnormal macrophage infiltration within the lesions. PF inhibits the macrophage-related rosacea-like inflammatory response through the SOCS3-ASK1-p38 pathway, demonstrating its potential application as a novel drug for rosacea therapy.

## Abstract 2

Supplemental Digital Content is available in the text

## 1 Introduction

Rosacea is a common chronic relapsing inflammatory skin condition that mostly affects the face. Its clinical manifestations include transient and recurrent flushing, persistent erythema, dilated capillaries, papules, and pustules. There are four common subtypes of rosacea, namely erythematotelangiectatic (ETR), papulopustular (PPR), phymatous (PhR), and ocular, which are all histologically characterized by granulomatous changes.^[1]^ The National Rosacea Society has now recognized a rosacea variant known as granulomatous rosacea, which is characterized by chronic, hard, painless reddish-brown cutaneous papules or nodules forming on relatively normal-appearing skin around the mouth and eyes and on the cheeks.^[2]^ Although the exact pathogenesis of rosacea is currently unclear, previous research has suggested that this chronic inflammatory disease has a certain genetic basis. Rosacea is caused by heightened vascular reactivity due to dysregulation of the innate immune system or can be triggered by various external factors, including the overgrowth of *Demodex* mites.^[3]^

The skin senses external stimuli through innate immune pattern recognition systems such as Toll-like receptors (TLRs), which induce cell responses and trigger inflammatory responses through microbial recognition.^[4]^ In response to environmental factors that trigger rosacea, including ultraviolet radiation, microbes, physical or chemical stimuli, and temperature changes, TLR2 is activated and abnormally high cathelicidin antimicrobial peptide (CAMP, known as LL37 in humans) levels are expressed in the skin of patients.^[5,6]^ Many studies have shown that various cells of the innate immune system, including macrophages, mast cells, and neutrophils, play a pivotal role in the pathogenesis of rosacea, with an increase in macrophages occurring in all subtypes of this condition. Buhl et al^[7]^ performed immunohistochemical (IHC) staining for CD68 on lesion tissues collected from patients with different rosacea subtypes and found strongly significant increases in CD68^+^ cells (i.e. macrophages) in all subtypes. The CD68^+^ cells were found to be vastly distributed in granulomatous areas and interfollicular areas in cases of papulopustular rosacea, whereas a perivascular diffuse pattern was mainly observed in cases of erythematotelangiectatic rosacea.

Many kinds of therapy are used to treat rosacea, including oral tetracycline, retinoic acid, azelaic acid, metronidazole, and laser. The core mechanisms underlying rosacea treatment are anti-inflammatory. Paeoniflorin (PF) is a compound extracted from *Paeonia lactiflora*, with demonstrated effectiveness in the treatment of multiple inflammatory diseases. Many *in vitro* studies have proven that PF possesses anti-inflammatory and immunosuppressive effects and can be used to treat rheumatoid arthritis, inflammatory bowel disease, and psoriasis.^[8,9]^ Although the precise mechanism of action has not been clearly elucidated, PF was shown to induce the expression of suppressor of cytokine signaling 3 (SOCS3), a negative regulator, thereby inhibiting the biological activity of the apoptosis signal-regulating kinase 1 (ASK1) and effectively alleviating postoperative pain. This effect was abolished by small interfering RNAs (siRNAs) targeting SOCS3.^[10]^ Another study showed that PF provided a remarkable renal protective effect in diabetic mice by suppressing inducible nitric oxide expression and the production of tumor necrosis factor-alpha, interleukin (IL)-1β, and monocyte chemoattractant protein-1 through the TLR4 signaling pathway, which in turn affected macrophage infiltration and activation.^[11]^ However, the effects of PF on rosacea and rosacea-like inflammatory responses have not been scientifically proven. Accordingly, this study investigated the role of macrophages in rosacea, as well as the influence of PF on lipopolysaccharide (LPS)-induced macrophage-related rosacea-like inflammatory responses and the underlying mechanisms. These findings might offer new insights into the pathogenic mechanisms contributing to the inflammatory response in rosacea and provide a theoretical basis for developing PF as a new treatment for this condition.

## 2 Materials and methods

### 2.1 Patient tissues

Tissue specimens of nine patients with clinically and histologically diagnosed granulomatous rosacea were obtained from the Department of Dermatology of The First Affiliated Hospital of Nanjing Medical University. The study was ethically approved by the Institutional Research Committee of the First Affiliated Hospital of Nanjing Medical University (2020-SRFA-082).

### 2.2 Hematoxylin and eosin (H&E) staining

Patient tissue specimens were fixed with formaldehyde, dehydrated with a graded alcohol series, embedded in paraffin, sectioned, dewaxed, and subjected to H&E staining. Stained sections were subsequently observed and photographed under an optical microscope.

### 2.3 IHC staining

Paraffin sections (5–μm-thick) were dewaxed, hydrated, treated with 3% H_2_O_2_ for 10 minutes, and washed three times (2 min each) with distilled water. The sections were placed in a container containing citrate buffer and heated in a microwave oven to maintain the liquid temperature in the container at 92 to 98°C for 10 to 15  minutes. After heating, the container was removed, cooled at room temperature for 10 to 20  minutes, and washed 3–5 times with phosphate-buffered saline (PBS). The sections were blocked using 5% normal goat serum (diluted with PBS) and incubated at room temperature for 20  minutes. After removal of the serum, drops of the following primary antibodies (appropriately diluted with PBS) were added and incubation was performed overnight at 4°C: anti-SOCS3 (1:1000, 52113 s) from Cell Signaling Technology (Beverly, MA); anti-LL37 (1:1000, ab180760) and anti-CD68 (1:1000, ab125212) from Abcam (Cambridge, MA, USA). Incubation with PBS alone served as a negative control. Subsequently, the sections were washed three times with PBS and shaken dry, and drops of appropriately diluted goat horseradish peroxidase (HRP)-conjugated secondary antibody (Millipore) were added for incubation at 37°C for 30 minutes or at room temperature for 1 hour. After washing with PBS and diethyl pyrocarbonate-treated water (Shanghai Generay Biotech, China), the sections were set aside for coloration. DAB was added dropwise to each section under a microscope, and the section was washed with water to terminate the staining process upon coloration. The section was counterstained for 4  minutes using hematoxylin, washed with water to remove unbound hematoxylin, and differentiated for 10 s using a 1% HCl-ethanol solution before washing with water again. After the sections had been dehydrated, cleared, and mounted with neutral resin using conventional methods, observation and image acquisition were performed under an optical microscope. IHC stains were semi-quantitatively analyzed and assigned immunoreactive scores (IRSs) by multiplying the percentage of positive cells by the staining intensity. Evaluation of the nuclear staining reaction was performed in accordance with the IRS proposed by Remmele and Stegner as follows: IRS = SI (staining intensity) × percentage of positive cells PP. SI was determined as follows: 0 is negative; 1, weak; 2, moderate; 3, strong. percentage of positive cells was determined as follows: 0 was defined as negative; 1, 10% positive cells; 2, 11% to 50% positive cells; 3, 51% to 80% positive cells; 4, more than 80% positive cells.^[12]^

### 2.4 Cell culture and grouping

The RAW 264.7 (2 × 10^5^) mouse monocyte line (ATCC TIB-71; American Type Culture Collection, Manassas, VA) was used as the macrophage line. The cells were cultured in 35-mm dishes with high-glucose Dulbecco modified Eagle medium (Thermo Fisher Scientific, Waltham, MA) and 10% fetal bovine serum (Gibco). When 70% confluence was reached (generation 3–4), randomization of the cells into the following three groups was performed for subsequent experiments: control, LPS model (LPS group), and PF+LPS groups. LPS (Sigma-Aldrich, St. Louis, MO, USA) was added to the latter two groups at the same time point, and cells in the PF+LPS group were pretreated with 10^−5^ mol/L PF (MedChemExpress, USA) for 4 hour and cultured for 12 hour after the addition of 1 μg/mL LPS. An equivalent volume of PBS was added to the control group. The cells were cultured in an incubator at 37°C with 5% CO_2_ prior to further experimentation.

### 2.5 Cytomorphological observations

After 12 hour of culture, the 3 cell groups (control, LPS, and PF+LPS groups) were observed under an inverted microscope (Ti-5 optical microscope, Nikon Japan) to detect morphological changes.

### 2.6 Measurement of cell proliferation

RAW 264.7 cells were cultured in a 96-well plate and treated with PF at different concentrations (10^−2^ to 10^−9^ mol/L) for 24 hour. Subsequently, the supernatant in each well was removed, and 10 μL Cell Counting Kit-8 (Dojindo China) solution and 100 μL culture medium were added. After incubation at 37°C with 5% CO_2_ for 1 hour, the absorbance of the cell suspensions was measured at 450 nm using an EnSight Multimode plate reader (PerkinElmer) to assess cell proliferation.

### 2.7 Western blotting

The cells were lysed in radioimmunoprecipitation assay buffer (containing 1% protease and phosphatase inhibitor), and the total protein content was quantitatively measured using the bicinchoninic acid assay. Protein (35 μg) was separated by electrophoresis using a 10% sodium dodecyl sulfate-polyacrylamide gel, transferred onto a polyvinylidene fluoride membrane, blocked with 5% non-fat milk for 2 h, and incubated with the primary antibody overnight at 4°C. After washing the polyvinylidene fluoride membrane using tris-buffered saline with 0.1% tween, immunoreactivity was measured using rabbit HRP-conjugated secondary antibody (1:5000; Millipore), and the beta-actin level was measured as a control. The following primary antibodies were used: anti-SOCS3 (1:1000, 52113 s), anti-p38 (1:1000, 8690 s), and anti-ASK1 (1:1000, 8662 s) from Cell Signaling Technology (Beverly, MA, USA); anti-TLR2 (1:1000, ab16894) anti-LL37 (1:1000, ab180760) from Abcam (Cambridge, MA); anti-β-actin from Santa Cruz Biotechnology (Santa Cruz, CA, USA). Imaging was performed using a ChemiDoc^TM^ XRS+ system (Bio-Rad, USA) and an HRP substrate (Luminata; Millipore), and the bands on the western blots were scored by scanning and quantitatively analyzing grayscale values using ImageJ (National Institutes of Health, Bethesda, MD).

### 2.8 Reverse transcription-quantitative polymerase chain reaction analysis

Using the PrimeScript RT reagent kit, 1 μg of RNA isolated from the RAW 264.7 cells was reverse-transcribed to cDNA with TRIzol reagent (Life Technologies, Carlsbad, CA, USA) and then amplified using SYBR green Premix Ex Taq II (Vazyme Biotech, Nanjing, China) with HiScript II Select qRT SuperMix (Vazyme Biotech) in an Applied Biosystems Real-Time PCR System (Life Technologies) under the following reaction conditions: initial denaturation, 95°C, 30 seconds; 40 cycles of 95°C, 5 s and 60°C, 30 second; annealing, 95°C, 15 seconds, 60°C, 1 minute, and 95°C for 15 seconds. The ΔCT method was adopted for data analysis, and *actin* was used as the reference gene for calculating the relative gene expression levels of the treatment groups. Table 1 shows the list of qPCR primers used in this study.

**Table 1:** List of qPCR primers for RAW 264.7 cells.

Primer target gene	Sequence (5′-3′)
Actin	F: CCAAAGGCTAACCGTGAA
	R: CGGAAGCGTAGAGGGAGA
*Camp*	F: GCTGTGGCGGTCACTATCAC
	R: TGTCTAGGGACTGCTGGTTGA
*Tlr2*	F: GCAAACGCTGTTCTGCTCAG
	R: AGGCGTCTCCCTCTATTGTATT
*P38*	F: CTGACCGACGACCACGTTC
	R: CTTCGTTCACAGCTAGGTTGC

### 2.9 RNA interference

SOCS3 siRNA and control siRNA were purchased from Santa Cruz Biotechnology (Santa Cruz, CA, USA). SOCS3 siRNA was a pool of three target-specific 19–25-nt siRNAs comprising the following: #1: sense CAGCAUCUUUGUCGGAAGATT, antisense UCUUCCGACAAAGAUGCUGTT; #2: sense GUAUGAUGCUCCACUUUAATT, antisense UUAAAGUGGAGCAUCAUACTT; #3: sense CCAAGUGUUGAACUUAGAATT, antisense UUCUAAGUUCAACACUUGGTT. Control siRNA was used as a negative control. For this, 10 μM siRNA dissolved in 330 μL Lipofectamine 2000 (Invitrogen, Carlsbad, CA) was added into the medium.

### 2.10 Statistical analysis

Statistical analysis of the experimental data was performed in SPSS 17.0 (SPSS Inc., Chicago, IL). The Student t-test was used for pairwise comparisons of quantitative data, and the homogeneity of variance test and one-way analysis of variance were used for comparing multiple groups. Two-tailed tests were adopted for all analyses and differences were considered statistically significant when *P* < .05. The final results were obtained from the data of three independent experiments.

## 3 Results

### 3.1 Profound infiltration of macrophages and inflammatory factors in rosacea tissue specimens

**Figure 1:**
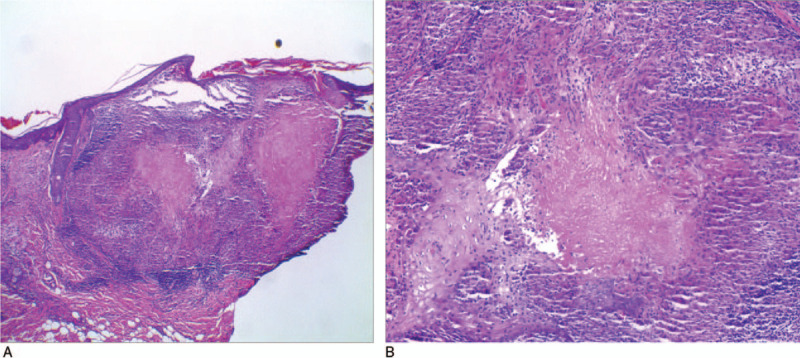
Histopathological manifestations of tissues from patients with rosacea (H&E staining, magnifications: A,:  × 40; B:  × 100).

**Figure 2:**
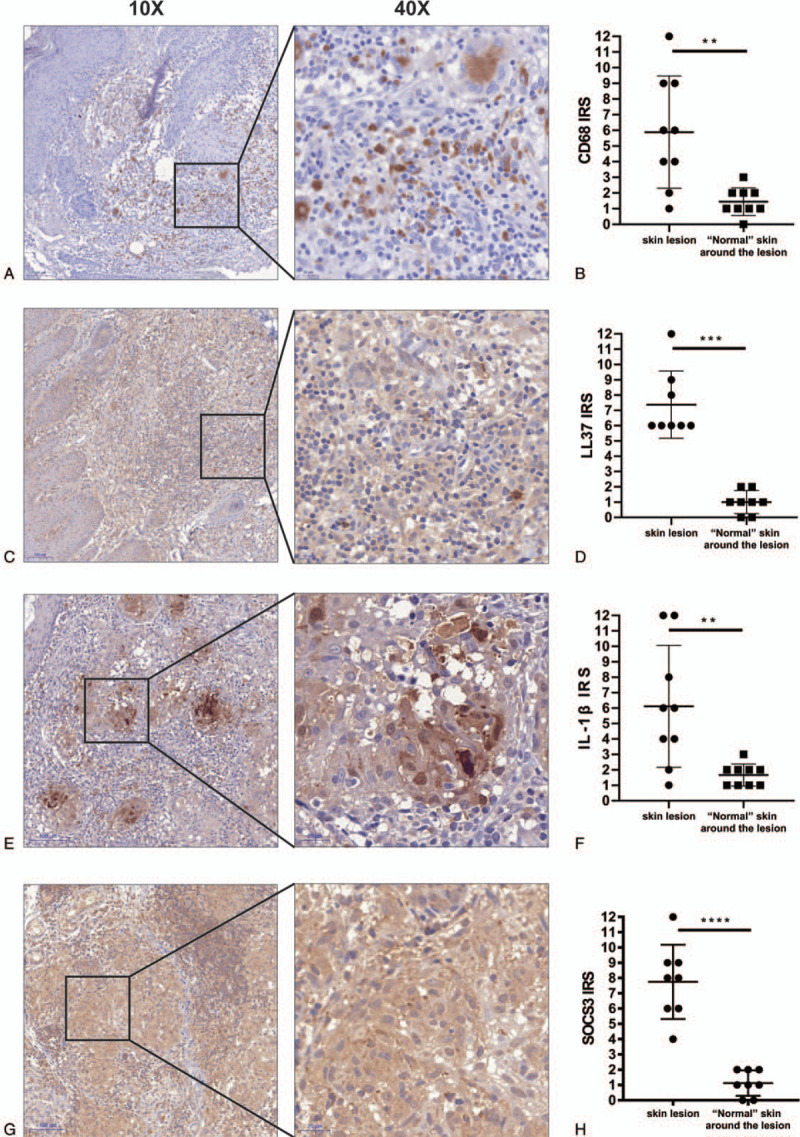
Immunohistochemical staining of tissues from patients with rosacea (A, B: CD68,  × 10,  × 40; D, E: LL37,  × 10,  × 40: G, H: IL-1β,  × 10,  × 40; J, K: SOCS3,  × 10,  × 40; C, F, I, L: Quantitative analysis of the positive staining rates of CD68, LL37, IL-1β and SOCS3, respectively, in lesions and peripheral areas using the immunoreactive score (IRS), N = 9, ^∗∗^*P* < .05; ^∗∗∗^p < 0.005; ^∗∗∗∗^*P* < .0005).

Under the microscope, based on HE-stained sections, a large number of inflammatory cells, mostly lymphocytes, in the dermis was found to have infiltrated the skin tissue, and epithelioid cell granuloma with a nodular distribution was observed (Fig. 1A-1B). CD68 immunohistochemical staining showed a large number of brown particles in the skin lesion area, which was significantly enhanced compared to that in the surrounding area of the skin lesion, suggesting a large amount of macrophage infiltration in the rosacea lesion area. Moreover, the expression of LL37, IL-1β, and SOCS3 in the skin lesion area was enhanced compared to that in the surrounding area. This indicates that the expression of these inflammatory factors was increased in the rosacea lesion area, and there was a statistical difference compared to that in the surrounding non-lesion area (Fig. 2A-2H)

### 3.2 Effect of PF on LPS-induced macrophage activation and synthesis of inflammatory factors

#### 3.2.1 Screening of appropriate PF concentration with no cytotoxicity

For different groups of RAW 264.7 cells 10^−9^ to 10^−2^ mol/L PF at different concentrations was added to the culture medium, and cells were cultured for 16 h to detect cell proliferation activity. We found that for RAW 264.7 cells co-cultured with 10^−2^ mol/L and 10^−3^ mol/L PF, cell proliferation activity was significantly lower than that of the blank control group (*P* < .05), but concentration lower than 10^−5^ mol/L PF had no significant effect on RAW 264.7 cell proliferation activity (see figure, Supplement figure, which illustrates influence of paeoniflorin at different concentrations on the proliferation of Raw264.7 cells)

#### 3.2.2 Effect of PF on LPS-induced macrophage activation

**Figure 3:**
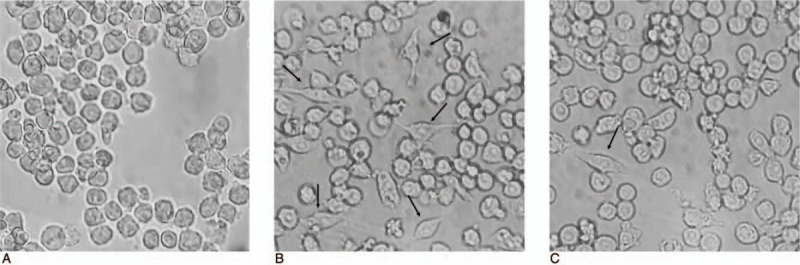
Morphology of RAW 264.7 cells in different groups under microscope (A, control; B, LPS; C, LPS+PF;  × 400).

Under the inverted microscope, the normal RAW 264.7 cells showed a uniform size with a circle or round shape, without obvious dendritic reactions. After LPS-induction, the cell body increased, pseudopodia protruded around the cell, and the cell shape did not change. This resulted in changes to a dendritic shape. The quantity of cells with dendritic changes among RAW 264.7 cells in the LPS+PF group was significantly reduced compared to that in the LPS group, and these were still mostly round or oval cells (Fig. 3A-3C).

#### 3.2.3 PF inhibits LPS-induced macrophage synthesis of LL37

**Figure 4:**
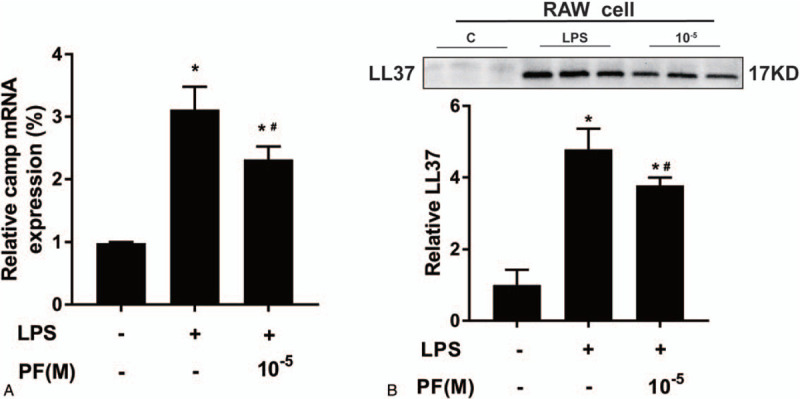
Paeoniflorin (PF) inhibits the LPS-induced expression of the *Camp* gene and LL37 protein in macrophages. (N = 3; compared with the control group, ^*∗*^*P* *<* *0.05*; compared with the LPS group, ^*#*^* P* *<* *.05*).

After the culture of RAW 264.7 cells with 1 μg/mL LPS, which was added to the medium for 12 hour, qRT-PCR results showed that the mRNA expression of the LL37 gene *camp* was significantly higher than that in the control group. However, in RAW 264.7 cells treated with 10^−5^ mol/L PF with added LPS, the expression of *camp* mRNA was higher than that in the control group but lower than that in the LPS group (*P* < .05). Western Blot analysis detected a similar trend in LL37 protein in the cell supernatant (Fig. 4A-4B)

**Figure 5:**
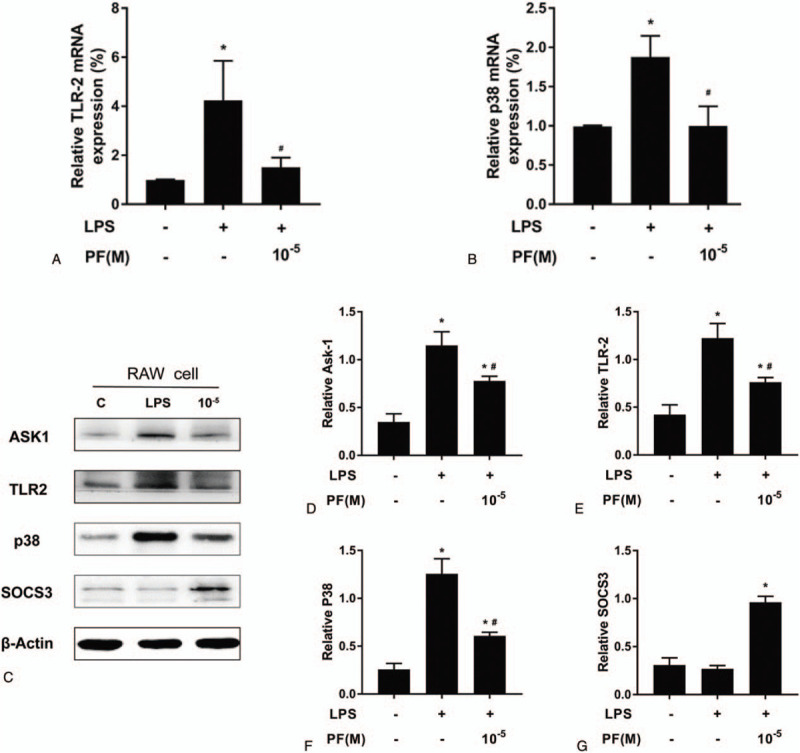
Paeoniflorin can inhibit the LPS-induced expression of Tlr2 and p38 genes in Raw264.7 cells and inhibit the LPS-induced increase in ASK1, TLR2, and p38 expression and decrease in SOCS3 expression in Raw264.7 cells. (A, B: tlr2 and p38 mRNA expression; C: SCOS3, ASK1, TLR2, and p38 protein expression; D-G: relative expression levels).

#### 3.2.4 Effect of PF on LPS-induced macrophage inflammatory factors

After the culture of RAW 264.7 cells with 1 μg/ml LPS, which was added to the medium for 12 h, the qRT-PCR results indicated that the expression of *Tlr2* and *p38* was significantly higher than that in the control group, and PF pretreatment could alleviate this change. For *Tlr2* and *p38* genes in the PF+LPS group, the expression was higher than that in the control group but lower than that in the LPS group. Western Blot analysis was performed to detect intracellular proteins in the control group, LPS group, and PF+LPS group, and the results were similar to the mRNA results. The expression of ASK1, TLR2, and p38 in RAW 264.7 cells of the LPS group increased, the expression of SOCS3 decreased, and pretreatment with PF alleviated LPS-induced changes in inflammatory factors. Thus, PF could inhibit the increase in ASK1, TLR2, and p38 expression and the decrease in SOCS3 expression in RAW 264.7 cells induced by LPS (*P* < .05).

### 3.3 SOCS3 siRNA inhibits the expression of inflammatory factors induced by PF in LPS-induced RAW 264.7 cells

**Figure 6:**
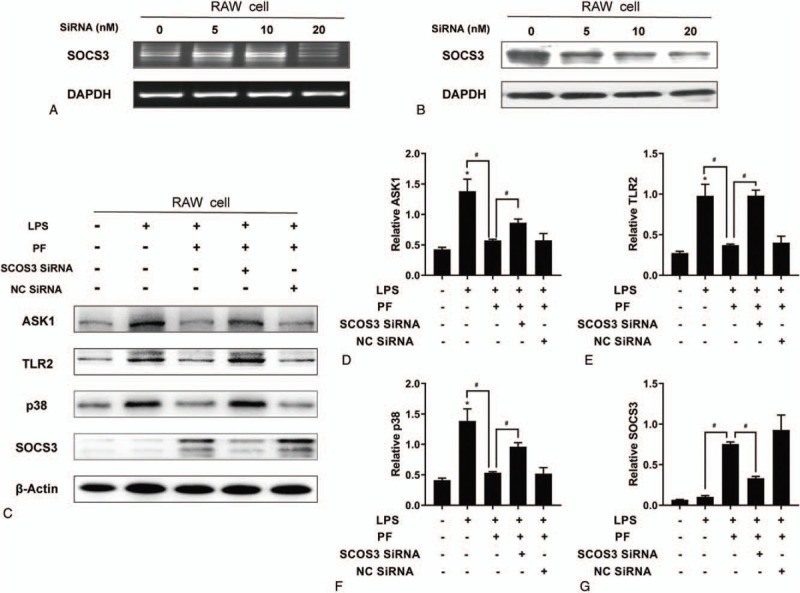
SOCS3 siRNA can inhibit PF to alleviate the LPS-induced inflammation in Raw264.7 cells. (A, B: plasmid concentration screening; C: SCOS3, ASK1, TLR2, and p38 protein expression; D-G: relative expression levels; compared with the control group, ^∗^*P* < .05; compared with the PF+LPS group, ^#^*P* < 0.05).

SOCS3 siRNA (20 nM) was used to interfere with SOCS3 expression. RAW 264.7 cells were treated with 0 or 10^−5^ mol/L PF or PF together with SOCS3 siRNA. The protein levels of SOCS3 and the aforementioned downstream molecules ASK1, TLR2, and p38 were detected by western blotting. The results showed that PF could increase the expression of SOCS3 and reduce the expression of ASK1, TLR2, and p38, whereas SOCS3 siRNA could inhibit these changes in RAW 264.7 cells that were induced by PF (Fig. 6).

## 4 Discussion

Rosacea is a common clinical chronic inflammatory skin disease. According to the typical manifestations, rosacea is often divided into four clinical subtypes, including ETR, PPR, hypertrophic type (PhR), and ocular type (ocular rosacea); however, due to the overlap in symptoms between subtypes and mutual transformation, in recent years, phenotypic description has gradually replaced clinical classification.^[13]^ The specific pathogenesis of rosacea is still unclear. It might be a chronic inflammatory disease dominated by multiple outcomes induced by innate immunity and abnormal vasomotor function resulting from a certain genetic background. There is literature indicating that rosacea is closely related to natural immune cells, including macrophages, mast cells, and neutrophils,^[14]^ and macrophages are important immune regulatory cells.

Buhl et al. found that there is significant macrophage and mast cell infiltration in rosacea lesions in three clinical subtypes of rosacea, namely ETR, PPR, and PhR.^[7]^ Smith JR et al. also found similar significant local macrophage infiltration in 20 cases of rosacea lesions accompanied by the expression of vascular endothelial growth factor (VEGF) and VEGF receptor 1 and high expression of VEGF receptor 2.^[15]^ Georgala S et al. reported similar results and speculated that the increased number of macrophages in rosacea lesions might be related to the excessive proliferation of *Demodex folliculata* in local areas.^[16]^ Recent studies have confirmed that the expression of disintegrin metalloprotease ADAM-like Decysin-1 is increased in the skin lesions of rosacea patients and LL37-induced rosacea-like mouse model skin lesions, and cytology experiments further confirmed that A disintegrin and metalloproteinase domain-like protein decysin-1 can participate in the inflammation associated with rosacea by promoting the classical activation of macrophages.^[17]^ Classically activated macrophages can be induced by LPS or Th1 cytokines and then secrete a large number of pro-inflammatory factors and chemokines, including IL-1β, IL-6, IL-12, and tumor necrosis factor-α, and participate in the inflammatory response.^[18]^ These results suggest that the activation of macrophages is closely related to the rosacea inflammatory response, and the molecules or components that inhibit the classical activation of macrophages might be potential drug targets for the treatment of rosacea.

Human antibacterial peptide LL37 is an important factor in the pathogenesis of rosacea. The intradermal injection of LL37 is a classic method to induce a rosacea mouse model.^[6]^ TLR2, which is highly expressed by keratinocytes in the skin lesions of rosacea patients, can increase the expression of LL37 by promoting the synthesis and activity of KLK5.^[19]^ TLRs can be expressed on the surface of keratinocytes and macrophages. TLR2 recognizes products of epidermal microorganisms, such as *Staphylococcus epidermidis* and *Propionibacterium acnes*, to promote inflammation through an increase in LL37, tumor necrosis factor-α, IL-1β, and other inflammatory factors, which in turn induces rosacea-related vasodilation, hyperplasia, and inflammation rash. Studies have confirmed that the expression of TLR2 and LL37 in the skin of rosacea patients is significantly increased. In our study, the pathology results of rosacea lesions showed significant inflammatory cell infiltration into the lesion area. The results of immunohistochemistry showed obvious macrophage infiltration, and the expression of the inflammatory factors LL37 and IL-1β was significantly increased, which was consistent with the results reported in previous literature.

The TLR2-mediated activation of p38 and extracellular signal-regulated kinase in response to streptococcal M1 protein,^[20]^*Staphylococcus epidermidis* LP01 lipopeptide,^[21]^ and other inflammatory and antibacterial reaction processes after skin stimulation are closely related. p38 protein kinase is the most important member of the mitogen activated protein kinases family, involved in controlling inflammation, and can further activate protein kinases and transcription factors related to inflammation. Wladis EJ et al. found that the expression of p38 and extracellular signal-regulated kinase in the eyelid skin tissue of patients with ocular rosacea was higher than that in the eyelid skin tissue of healthy people.^[22]^ Yamasaki et al^[6]^ and others found that LL37 can promote rosacea skin inflammation, whereas the inhibition of p38 can selectively inhibit the LL37-induced keratinocyte synthesis of IL-36ɣ.^[23]^ ASK1 is a member of the mitogen-activated protein kinase kinase (MAP3K) family. Reactive oxygen species can activate p38 via ASK1 and induce macrophages to express human β-defensins (hβD) 1–3 and LL37 to promote inflammation.^[24]^ These results suggest that TLR2, ASK1, and p38 play an important role in antibacterial peptide-mediated skin inflammation in rosacea. SOCS3 is a common inhibitory signal-regulatory protein that participates in many important signaling pathways, such as the negative feedback regulation of mitogen activated protein kinases. However, a variety of cytokines, such as IL-2, interferon-γ, and IL-6, can promote the expression of SOCS3 via feedback.^[25,26]^

PF is a monoterpene glycoside compound, the main component of the total glycosides of *Paeonia lactiflora*, and has certain anti-inflammatory and immunosuppressive effects. Studies in psoriasis model mice^[27]^ found that PF can reduce the infiltration of neutrophils and macrophages into psoriatic lesions and thereby reduce the expression of related inflammatory factors. Our cytology experiments found that PF pretreatment could alleviate the increase in LPS-induced macrophage synthesis of TLR2, ASK1, and p38, and the decrease in SOCS3, suggesting that PF can inhibit LPS-induced macrophage inflammation. Through rosacea histopathological analysis, we found that the expression of SOCS3 in the rosacea lesion area was significantly higher than that in the surrounding area. It is speculated that this might be due to the increase in multiple inflammatory factors in the rosacea lesions and the feedback-mediated promotion of the expression of the anti-inflammatory factor SOCS3.

Our study further verified that there is high macrophage infiltration into rosacea lesions, as well as enhanced expression of the inflammatory factors LL37, IL-1β, and SOCS3. At the cytological level, we initially explored the effect of PF on LPS-induced macrophage inflammation. It was found that the inhibitory effect of PF was achieved by slowing the increase in TLR2, ASK1, and p38 expression and the decrease of SOCS3 expression. Moreover, the SOCS3 siRNA experiment results further confirmed that the effect of PF on macrophage inflammation was achieved through the ASK1-p38 pathway. However, the experiment also had certain deficiencies. For example, we need to further verify our results in a rosacea mouse model.

In summary, rosacea is closely related to macrophages and inflammatory factors. PF can inhibit macrophage inflammation by affecting the SOCS3-ASK1-p38 pathway and might be a potential drug choice for the treatment of rosacea.

## Author contributions

**Conceptualization:** Zijing Liu.

**Data curation:** Zijing Liu, Jiawen Zhang, Peiyu Jiang, Yunyi Liu.

**Formal analysis:** Jiawen Zhang, Zhi Yin, Yunyi Liu, Yixuan Liu, Xiaoyan Wang, liang hu.

**Methodology:** Zhi Yin, liang hu, Wentao liu, Yang Xu.

**Project administration:** Wentao liu, Yang Xu.

**Resources:** Yang Xu.

**Writing – original draft:** Zijing Liu.

**Writing – review & editing:** Yang Xu.

## Supplementary Material

Supplemental Digital Content
