# Supplementary figures and images for: Paeoniflorin inhibits the macrophage-related rosacea-like inflammatory reaction through the suppressor of cytokine signaling 3-apoptosis signal-regulating kinase 1-p38 pathway
Source: Medicine (Baltimore). 2021 Jan 22;100(3):e23986. doi: 10.1097/MD.0000000000023986 (PMC7837818; doi:10.1097/MD.0000000000023986)

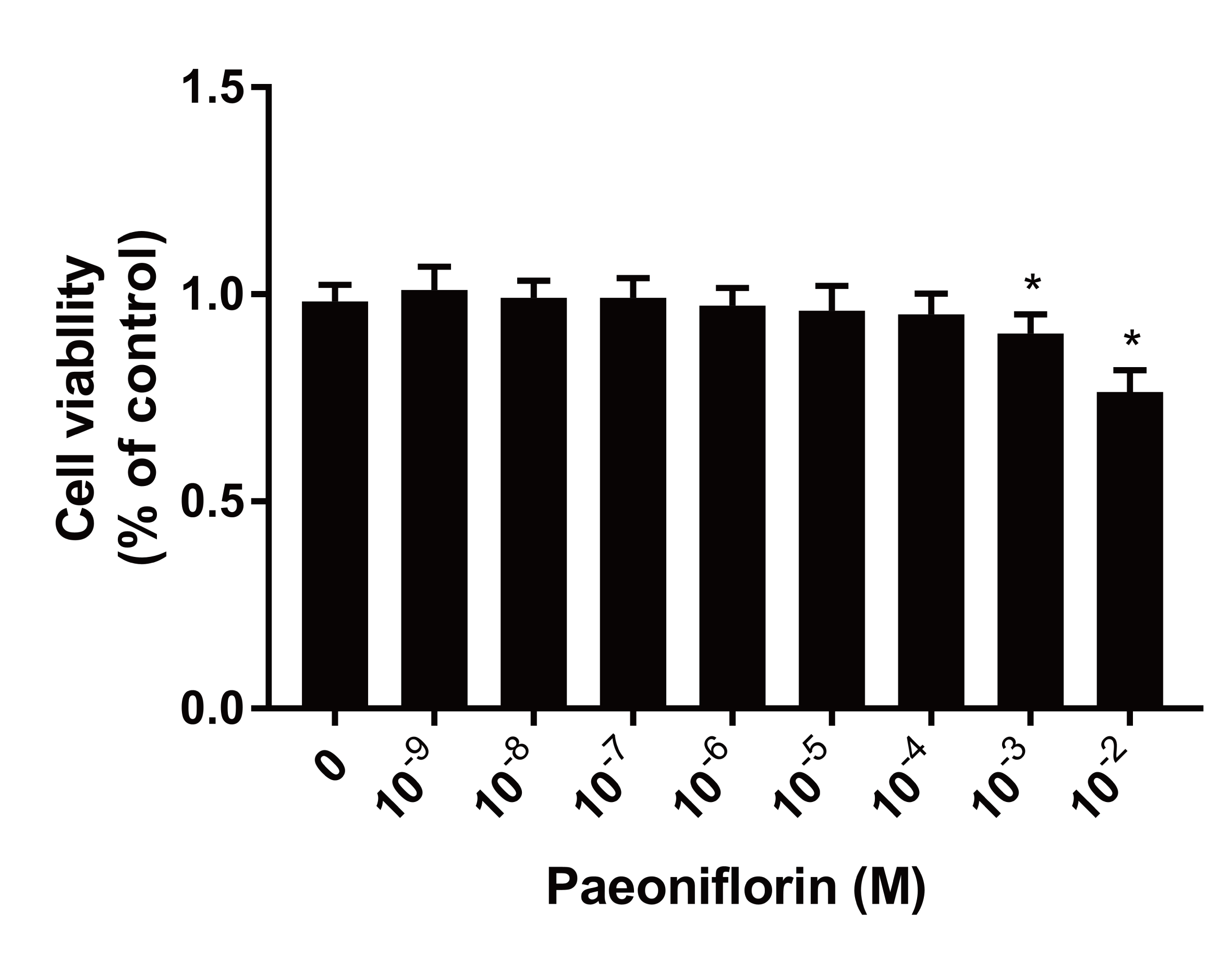

Supplement: Supplemental Digital Content [file medi-100-e23986-s001.tif]
